# Authentication and Quantitation of Fraud in Extra Virgin Olive Oils Based on HPLC-UV Fingerprinting and Multivariate Calibration

**DOI:** 10.3390/foods7040044

**Published:** 2018-03-21

**Authors:** Núria Carranco, Mireia Farrés-Cebrián, Javier Saurina, Oscar Núñez

**Affiliations:** 1 Department of Chemical Engineering and Analytical Chemistry, University of Barcelona, Martí i Franqués, 1-11, E08028 Barcelona, Spain; nuria.carranco.cruz@gmail.com (N.C.); mirefarres28@gmail.com (M.F.-C.); xavi.saurina@ub.edu (J.S.); 2 Research Institute in Food Nutrition and Food Safety, University of Barcelona, Recinte Torribera, Av. Prat de la Riba 171, Edifici de Recerca (Gaudí), Santa Coloma de Gramenet, E08921 Barcelona, Spain; 3 Serra Húnter Fellow, Generalitat de Catalunya, Rambla de Catalunya 19-21, E08007 Barcelona, Spain

**Keywords:** high performance liquid chromatography, UV detection, multivariate calibration, food authentication, olive oils, fraud quantitation

## Abstract

High performance liquid chromatography method with ultra-violet detection (HPLC-UV) fingerprinting was applied for the analysis and characterization of olive oils, and was performed using a Zorbax Eclipse XDB-C8 reversed-phase column under gradient elution, employing 0.1% formic acid aqueous solution and methanol as mobile phase. More than 130 edible oils, including monovarietal extra-virgin olive oils (EVOOs) and other vegetable oils, were analyzed. Principal component analysis results showed a noticeable discrimination between olive oils and other vegetable oils using raw HPLC-UV chromatographic profiles as data descriptors. However, selected HPLC-UV chromatographic time-window segments were necessary to achieve discrimination among monovarietal EVOOs. Partial least square (PLS) regression was employed to tackle olive oil authentication of Arbequina EVOO adulterated with Picual EVOO, a refined olive oil, and sunflower oil. Highly satisfactory results were obtained after PLS analysis, with overall errors in the quantitation of adulteration in the Arbequina EVOO (minimum 2.5% adulterant) below 2.9%.

## 1. Introduction

Olive oil is one of the main ingredients in the Mediterranean diet, and over the past few years its consumption has also spread outside the Mediterranean basin. This is due to both its particular taste and remarkable nutritional properties. In fact, virgin olive oil (VOO) is the primary fat source in the Mediterranean diet, and it is obtained either by mechanical or direct pressing of the pulp of the olive fruit (*Olea europaea* L.). The olives, after being crushed to form pomace, are homogenized and pressed. VOO is not subjected to any treatment other than washing, decantation, centrifugation, and filtration [1]. Extra-virgin olive oil (EVOO) is considered to be the highest quality olive oil, and is characterized by high levels of beneficial constituents [2]. Apart from the natural unrefined state, olive oils of lower quality known as refined olive oils (ROOs) are also produced. For example, an extracted oil with poor sensory characteristics, high free fatty acid content, or peroxide values exceeding the established limits, is unfit for human consumption. This oil is then classified as lampante oil and needs to be refined. Additionally, pomace oils are solvent-extracted from olive pomace and also undergo refining [1,3]. Triglycerides are the major constituents of EVOOs, representing more than 98% of the total weight. The fatty acid composition of these triglycerides involves saturated fatty acids (11%), around 80% monounsaturated fatty acids (oleic acid being the most important feature of olive oils in comparison to other vegetable oils), and polyunsaturated fatty acids (9%). Furthermore, EVOO also contains antioxidant and anti-inflammatory compounds such as squalene, phytosterols, and highly bioavailable polyphenols and phenolic acids belonging to different families [2,4,5,6,7,8,9]. These compounds provide important beneficial health effects.

The beneficial characteristics of EVOOs and their high quality with respect to other edible oils make them an expensive product, susceptible to being adulterated with oils produced from cheaper fruits or seeds [10], or even with other lower quality olive oils. Moreover, lately there have been suspicions among consumers and administrations that in some cases producers have labeled virgin olive oils or even refined ones as EVOOs in order to increase profits. A comprehensive academic food fraud study spanning 30 years in the Journal of Food Science showed that olive oil was the single most commonly referenced adulterated food of any type in scholarly articles from 1980 to 2010 [11]. This is a relevant problem not only because of the financial issues affecting olive oil-producing countries such as Spain or Italy, but also in terms of health and consumer protection concerns. However, identification of some fraud is especially difficult, or can be time- and solvent-consuming when using traditional methodologies for the identification of adulteration, e.g., when the cheaper oil is added to EVOO at a level lower than a certain percentage. Therefore, the reduction of the current levels of fraud in olive oils requires the constant development of new analytical techniques for the detection of possible adulteration. In this regard, the study of compositional differences among olive oils and other edible vegetable oils which could be used as adulterants (e.g., because of their low cost) has been the basis for authentication approaches.

The types of polyphenols and phenolic acids and their concentration levels in olive oils depend on multiple parameters such as climate conditions, water resources, growing area, cultivation techniques, soil management, and the degree of maturation of the olives. Moreover, the oil production method (malaxation and extraction) can also affect the final quantities of these compounds [12,13,14,15]. As a result, polyphenol and phenolic acid content can be exploited as a source of analytical data to establish vegetable oil product classifications and to achieve product authentication [16,17], thus preventing fraud by detecting and quantifying product adulteration.

Several analytical methodologies have been employed in the determination of polyphenols in olive oils [18,19]. One of the most critical points is the extraction procedure, which is frequently addressed by liquid–liquid extraction (LLE) [20,21,22,23,24] and solid phase extraction (SPE), mainly using diol-bonded SPE cartridges [8,22,24,25,26,27,28]. Capillary electrophoresis (CE), liquid chromatography (LC), and gas chromatography (GC) are among the separation techniques usually employed for the determination of polyphenols [18,19,29]. However, the necessity for a derivatization step prior to GC separation [30] and the low sensitivity of CE methods when on-column UV detection is employed [20,24,31] make LC the technique of choice nowadays for the determination of polyphenols and phenolic compounds in olive oils. With respect to detection, electrochemical detection (ECD) [26], fluorescence [32], ultra-violet (UV) detection [8,24,32,33,34], mass spectrometry (LC-MS) [35] or tandem mass spectrometry (LC-MS/MS) [24,27,28,36] are currently used. Recently, due to the complexity of edible oil samples and the high structural variability of polyphenols, liquid chromatography coupled to high resolution mass spectrometry (LC-HRMS) appears to be the best technique for the identification and determination of phenolic compounds in vegetable oils [22,25], although these techniques are still expensive for many food control laboratories. Lately, interest in the classification and characterization of olive oils with respect to olive varieties, as well as in the identification and quantitation of fraud to guarantee product authentication, has been increasing, and several studies devoted to these topics can be found in the literature [21,37,38,39,40].

The aim of the present work was to develop a simple, cheap, and reliable high performance liquid chromatography method with UV detection (HPLC-UV) for the generation of polyphenolic fingerprints in the classification and authentication of olive oils. Data corresponding to the HPLC-UV polyphenolic fingerprints recorded at different wavelengths were considered as a source of potential descriptors to be exploited for the characterization and classification of edible oils (olive, sunflower, soy, and corn oils) and olive oils from different olive cultivars (Arbequina, Picual, Hojiblanca, and Cornicabra) by exploratory principal component analysis (PCA). Finally, Arbequina EVOO was adulterated with different amounts (2–85%) of Picual EVOO, a commercial low quality ROO, and a sunflower oil. The HPLC-UV data was evaluated by means of partial least squares (PLS) regression for authentication purposes as well as for the quantification of adulterant content.

## 2. Materials and Methods

### 2.1. Chemicals and Standard Solutions

Unless specified, analytical grade reagents were used. Methanol (Chromosolv™ for HPLC, ≥99.9%), hexane, and formic acid (≥98%) were obtained from Sigma-Aldrich (St. Louis, MO, USA), and ethanol (absolute) from VWR International Eurolab S.L. (Barcelona, Spain). 

Water was purified using an Elix 3 coupled to a Milli-Q system (Millipore, Bedford, MA, USA) and was filtered through a 0.22-µm nylon membrane integrated into the Milli-Q system.

### 2.2. Instrumentation

An Agilent 1100 Series HPLC instrument equipped with a G1311A quaternary pump, a G1379A degasser, a G1392A autosampler, a G1315B diode-array detector, and a computer with the Agilent Chemstation software (Rev. A 10.02), all from Agilent Technologies (Waldbronn, Germany), was employed to obtain the HPLC-UV chromatographic fingerprints for the PCA and PLS studies. Chromatographic separation was carried out in reversed-phase mode by using a Zorbax Eclipse XDB-C8 column (150 × 4.6 mm i.d., 5 µm particle size) also provided by Agilent Technologies. Formic acid (0.1%, *v*/*v*) aqueous solution (solvent A) and methanol (solvent B) were used as mobile phase to stablish the gradient elution as follows: 0–2 min at 10% B (initial conditions); 2–4.5 min linear gradient from 10% B to 25% B; 4.5–7 min at 25% B; 7–22 min linear gradient from 25% B to 90% B; 22–24 min at 90% B; 24–25 min back to initial conditions at 10% B; and 25–30 min at 10% B for column equilibration. A mobile phase flow-rate of 1 mL min^−1^ and an injection volume of 10 µL were employed. Photodiode array (PDA) acquisition from 190 to 600 nm was performed to register UV spectra and to guarantee peak purity when necessary. HPLC-UV fingerprints for PCA and PLS analysis were obtained by direct UV absorption detection at 257, 280, and 316 nm.

### 2.3. Samples and Sample Treatment

Two sets of vegetable oil samples belonging to different trademarks and purchased from markets in Barcelona (Spain) were used in this work. The first set of samples consisted of 72 vegetable oils distributed as follows: 47 olive oils, 16 sunflower oils, 2 corn oils, 2 soy oils, and 5 vegetable oils produced from mixtures of seeds (3 sunflower/corn oils and 2 sunflower/soy oils). Among the 47 olive oils belonging to this first set of samples, 10 were obtained from Arbequina olives and 5 from Picual olives. No information regarding the olive cultivar of other 32 olive oil samples was available. The second set of samples consisted of 66 EVOO samples as follows: 23 from Arbequina olives, 19 from Picual olives, 12 from Hojiblanca olives, and 12 from Cornicabra olives. Additionally, several refined olive oil samples were also employed for the adulteration studies.

Sample treatment was carried out following a previously described method with some modifications [28]. For that purpose, 2.00 g of oil were weighed into a 15 mL polytetrafluoroethylene (PTFE) tube (Serviquimia, Barcelona, Spain), and extracted with 2 mL of an ethanol:water (70:30, *v*/*v*) solution by shaking vigorously for 2 min using a vortex (Stuart, Stone, UK). The extract was then centrifuged for 5 min at 3500 rpm (Rotanta 460 RS centrifuge, Hettich, Tuttlingen, Germany). In order to facilitate the quantitative recovery of the extract solution, the PTFE tube was then frozen for 24 h at −18 °C, and the corresponding extract transferred into another 15 mL PTFE tube for further clean-up. Defatting was performed with 2 mL of hexane, shaking vigorously for 2 min in a vortex, and centrifuging for 5 min at 3500 rpm. Finally, the aqueous ethanolic extracts were transferred into 2 mL injection vials to be analyzed with the proposed HPLC-UV method.

Besides, a quality control (QC) consisting of a mixture of 50 µL of each ethanolic aqueous sample extract was prepared to evaluate the repeatability of the method and the robustness of the chemometric results. 

For authentication studies by PLS regression, three cases were considered in which an Arbequina EVOO sample was adulterated with different amounts (from 2.5 to 85%) of a Picual EVOO sample, a ROO sample, or a sunflower oil sample, respectively. Hence, apart from those pure extracts (three samples each), mixtures of the Arbequina EVOO sample and the adulterant oil were as follows: 85% adulterant (3 samples), 80% adulterant (3 samples), 60% adulterant (3 samples), 55% adulterant (3 samples), 50% adulterant (8 samples), 40% adulterant (3 samples), 30% adulterant (3 samples), 20% adulterant (3 samples), 12% adulterant (3 samples), 10% adulterant (3 samples), 7% adulterant (3 samples), 5% adulterant (3 samples), 3% adulterant (3 samples), and 2.5% adulterant (3 samples), for each adulterant oil employed.

### 2.4. Data Analysis

SOLO chemometric software from Eigenvector Research was used for calculations with PCA and PLS regression [41]. A detailed description of the theoretical background of these methods is given elsewhere [42]. 

Data matrices to be treated by PCA consisted of the HPLC-UV chromatographic fingerprints obtained at different acquisition wavelengths (257, 280, and 316 nm). HPLC-UV chromatograms were pretreated to improve the data quality while minimizing solvent and matrix interferences, peak shifting, and baseline drifts (for additional details see [43]). In some cases, segmented HPLC-UV fingerprints were employed to improve PCA classification. Scatter plots of scores and loadings of the principal components (PCs) were used to investigate the structure of maps of samples and variables, respectively.

The percentage of the oil used for adulteration (Picual EVOO, ROO, or sunflower oil) in the adulterated Arbequina EVOO samples analyzed was quantified using PLS. Samples available were distributed among training and test sets as follows. Training set: 100% adulterant (3 samples), 80% adulterant (3 samples), 60% adulterant (3 samples), 50% adulterant (8 samples), 40% adulterant (3 samples), 20% adulterant (3 samples), 10% adulterant (3 samples), 5% adulterant (3 samples), 2.5% adulterant (3 samples), and 100% Arbequina EVVO (3 samples). The remaining samples considered as unknown (85% adulterant, 55% adulterant, 30% adulterant, 12% adulterant, 7% adulterant and 3% adulterant, 3 samples each) were used for validation and prediction purposes. For both training and test steps, X-data matrices consisted of the HPLC-UV chromatographic fingerprints and the Y-data matrices contained the oil adulteration percentages.

## 3. Results and Discussion

### 3.1. Exploratory Studies by Principal Component Analysis

As a first study, the characterization and classification of olive oils with respect to other edible vegetable oils (sunflower, corn, and soy oils, as well as mixtures of them) was attempted by using raw HPLC-UV chromatographic profiles (i.e., absorbance over time) as analytical data for PCA. Reversed-phase HPLC-UV chromatographic fingerprints were obtained by employing a previously developed method in a Zorbax Eclipse XDB-C8 column under gradient elution using methanol and 0.1% aqueous formic acid solutions as mobile phase [21]. HPLC-UV chromatographic fingerprints were studied at three acquisition wavelengths: 257, 280, and 316 nm. The reproducibility of the extraction step and its influence on the PCA results was studied elsewhere [21]. Relative standard deviation (RSD%) values of polyphenol features ranged from 0.3 to 8.3% depending on the levels of components. Replicate samples have similar PCA scores, thus indicating that extraction variabilities did not significantly affect the descriptive and quantitative performance of chemometric models. The variability of chromatographic runs was evaluated from the repetitive injections of the QC (every 10 sample injections) throughout the series. In this way, the influence of peak shifting and/or baseline drift on the PCA models was deduced from trends observed in the scores plot. When QCs were not clustered randomly due to systematic deviations, corrective data pretreatments such as peak alignment and baseline subtraction were applied [43].

**Figure 1:**
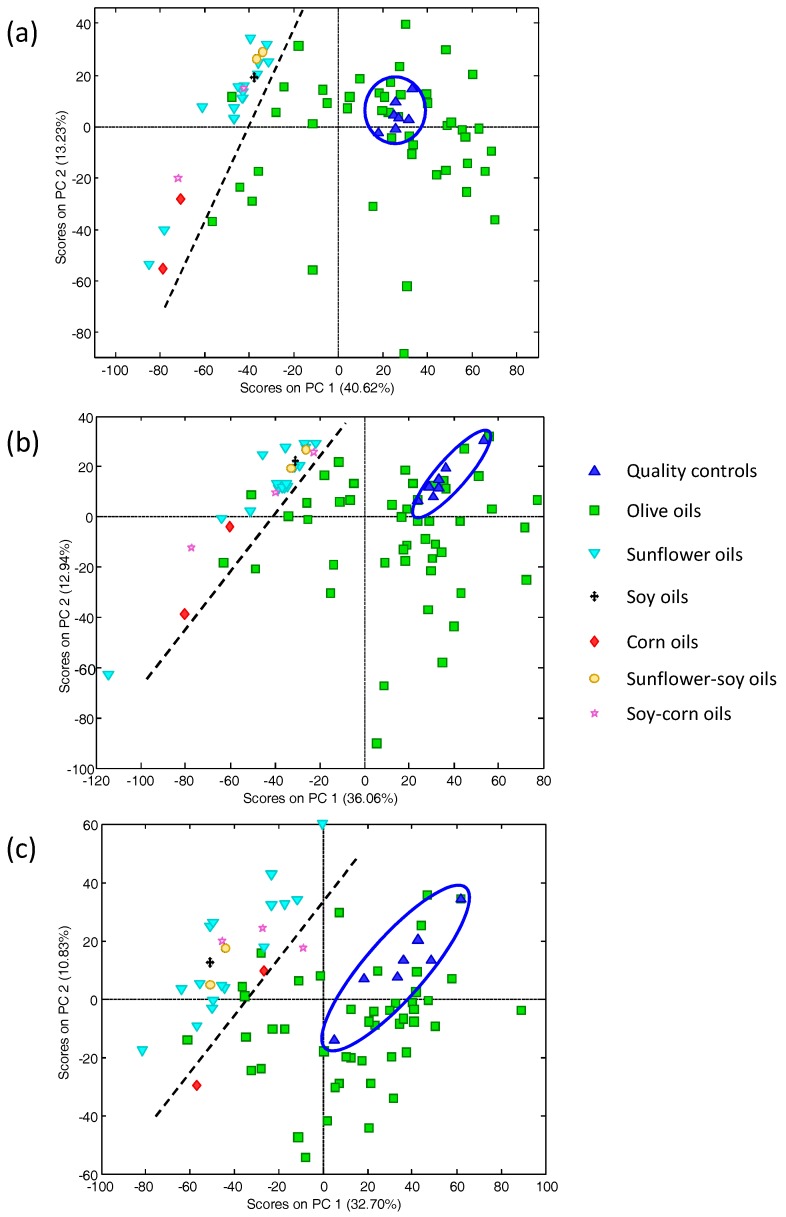
Scatter score plots (PC1 vs. PC2) when processing raw HPLC-UV (High performance liquid chromatography method with ultra-violet detection) chromatographic fingerprints of 47 olive oils and 25 other fruit seed oils (sunflower, corn, soy, and some mixtures of them) registered at (**a**) 257 nm; (**b**) 280 nm; and (**c**) 316 nm. Ellipse is grouping the quality controls.

A first set of samples consisting of 72 commercially available vegetable oils was employed. The ethanolic extracts as well as the QCs (see Experimental section) were randomly analyzed with the proposed method, and the obtained HPLC-UV fingerprints subjected to PCA. At first, the performance of the resulting models was quite limited due to experimental variability of chromatograms, displaying peak shifting and baseline drifts that affected the distribution of samples. For this reason, chromatogram pre-processing was recommendable to improve the analytical performance of PCA models. Thus, data matrices were autoscaled and then subjected to PCA. Figure 1 shows the score plots of PC1 vs. PC2 when processing HPLC-UV chromatographic fingerprint data registered at (a) 257 nm; (b) 280 nm; and (c) 316 nm.

As can be seen, in general QCs appeared in compact groups, demonstrating a good repeatability and robustness of results, with the exception of the dataset at 316 nm (Figure 1c) where a relatively higher dispersion on QCs was observed. Regarding sample characterization and classification, a good differentiation between olive oils against the other oil samples was obtained. In all cases, olive oils tend to be distributed to the right part of the plots across PC1, a component that seems to be related to the polyphenolic content. All other fruit or seed oils apart from olive ones were located to the left part of the PC1 vs. PC2 plot, and a certain discrimination among samples was observed although this was not enough to achieve reasonable clustering according to the fruit/seed of origin (sunflower, soy, or corn). Nevertheless, the results obtained after this exploratory study with PCA were very promising with regard to the authentication of olive oil samples. Regarding the set of 47 EVOO samples, Arbequina and Picual oils were mainly distributed in predominant areas. Oils of non-declared cultivars were basically located in intermediate positions in agreement with their expected blended nature. The HPLC-UV chromatographic fingerprint data was refined by considering only the information of several chromatographic time segments as a way to improve the differentiation and classification of oil samples. For that reason, chromatographic data, at the three wavelengths evaluated, was autoscaled and the following time windows were selected: (1) a 5–8 min segment; (2) an 11–15 min segment; and (3) an 11–20 min segment, as well as the data matrices combining two time windows: (1) 5–8 min + 11–15 min segments; and (2) 5–8 min + 11–20 min segments, and were subjected to PCA. From the direct observation of the HPLC-UV chromatographic profiles obtained from the analyzed samples (see as example Figure S1 in the Supplementary Materials depicting the HPLC-UV fingerprints at 280 nm of two olive oils and a sunflower oil) the studied time segments seem to have, a priori, a certain differentiation, and PCA results showed discrimination between olive oils and other vegetable oils (see two selected examples in Figure S2 in the Supplementary Materials). None of the models evaluated allowed a better classification than the one obtained after considering the full HPLC-UV chromatographic fingerprints (Figure 1).

**Figure 2:**
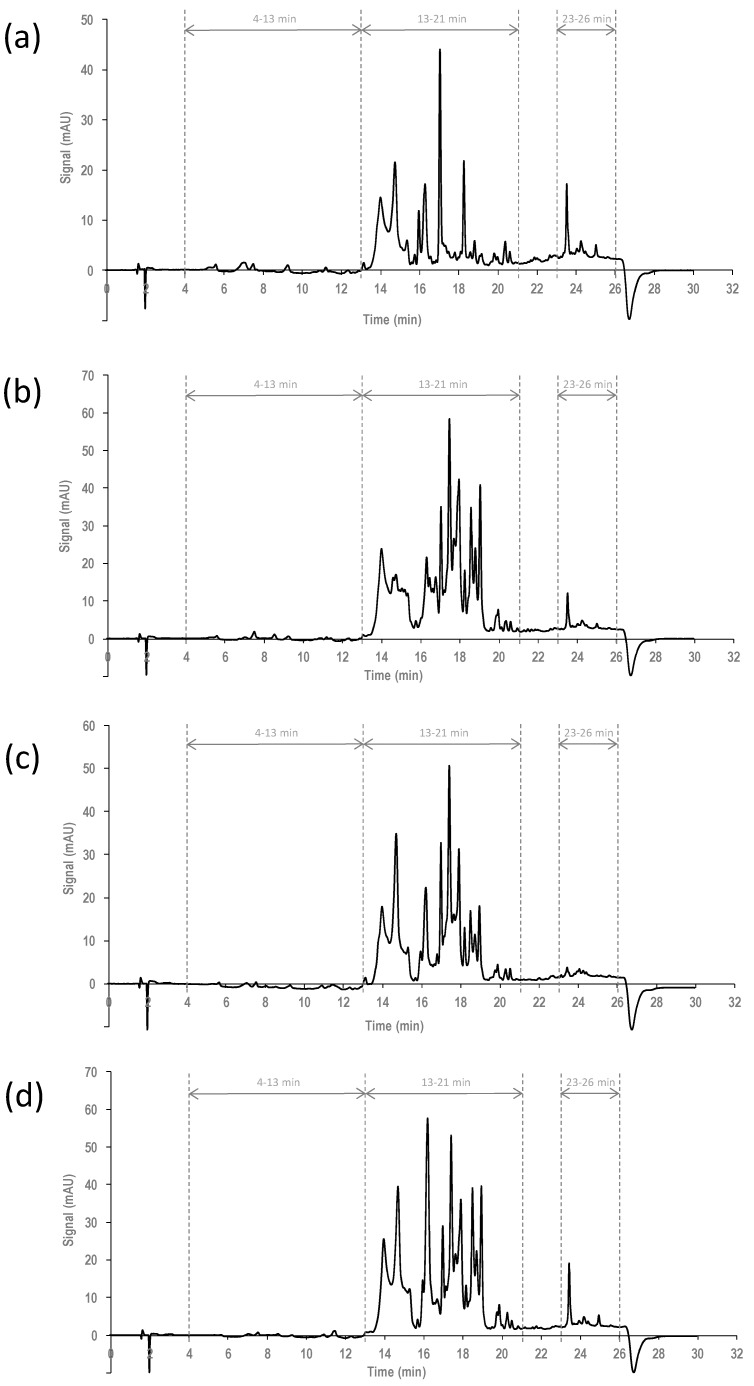
HPLC-UV chromatographic fingerprint registered at 257 nm for four extra-virgin olive oil (EVOOs) obtained from (**a**) Arbequina; (**b**) Picual; (**c**) Hojiblanca; and (**d**) Cornicabra monovarietal olive cultivars.

In a second study, the characterization and classification of EVOOs with respect to the olive cultivar of origin (Arbequina, Picual, Hojiblanca, and Cornicabra) was attempted by using the raw HPLC-UV chromatographic profiles and PCA. For that purpose, a new set of samples was analyzed, consisting of 66 commercially available EVOOs distributed as follows: 23 from Arbequina olives, 19 from Picual olives, 12 from Hojiblanca olives, and 12 from Cornicabra olives. As an example, Figure 2 shows the HPLC-UV chromatographic fingerprints registered at 257 nm from (a) Arbequina; (b) Picual; (c) Hojiblanca; and (d) Cornicabra monovarietal EVOOs. In general, the HPLC-UV chromatographic profiles are different depending on the cultivar, as expected, although some similarities can be observed. These profiles can be divided into three time-window segments. The first one, from 4 to 13 min, is characterized for the presence of very few extracted compounds with low signal intensities. In the second one, from 13 to 21 min, most of the extracted compounds when using ethanol:water (70:30, *v*/*v*) solution are eluted. It is characterized for richer profiles with slight differences depending on the olive cultivar of origin. For instance, if we consider some selected time-window sections within this segment, it is possible to see that from 13 to 16 min, a similar signal profile was obtained for the Arbequina, Hojiblanca, and Cornicabra cultivars (with only slight differences in signal intensity) in comparison to the Picual cultivar. From 16 to 17 min it seems that three different signal profiles can be observed: one obtained for Hojiblanca and Cornicabra, and the characteristic ones for Arbequina and Picual, respectively. In contrast, the time-window section from 17 to 21 min clearly provides a very different UV chromatographic fingerprint for Arbequina in comparison to the other three cultivars, which seem to differ only in signal intensity. Finally, a last time-window segment from 23 to 26 min can be considered, the Hojiblanca cultivar being the one showing a characteristic profile in comparison to the other three olive cultivars evaluated. The differences observed in both the chromatographic patterns and the signal relative abundances seem to be representative of each olive variety so that they can be exploited as potential chemical descriptors to achieve classification and authentication of oils by chemometric methods.

Thus, raw HPLC-UV chromatograms of the 66 EVOOs were evaluated as the first PCA model, and the obtained score plots (PC1 vs. PC2) at the three evaluated wavelengths are depicted in Figure S3 (Supplementary Materials). Discrimination of EVOOs regarding the olive cultivar was not achieved successfully under these conditions. Most of the Arbequina EVOOs seem to be clustered together and separated from the other EVOOs at the bottom (data registered at 257 and 280 nm) and at the top (data registered at 316 nm) areas of the score plots, in agreement with the fact that Arbequina EVOOs clearly show the most different raw HPLC-UV chromatographic fingerprints (Figure 2a) in comparison to the other three olive cultivars.

**Figure 3:**
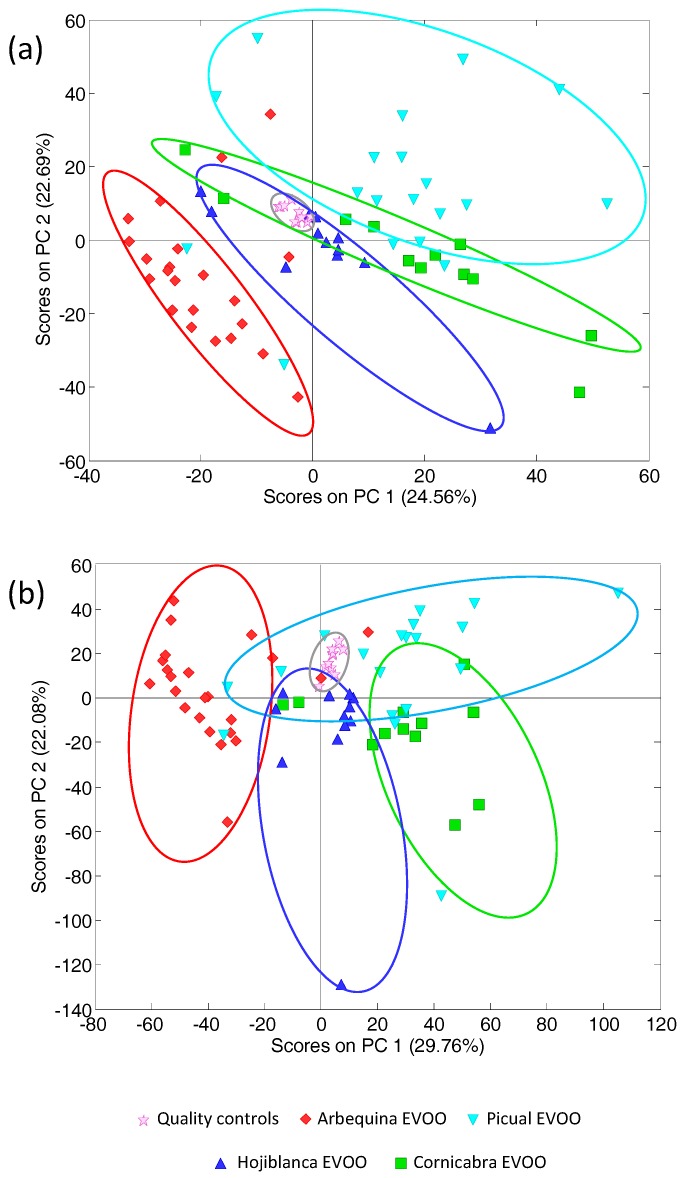
Principal component analysis (PCA) results (scatter score plot of PC1 vs. PC2) in the analysis of 66 EVOOs obtained from monovarietal olive cultivars by employing as data: (**a**) HPLC-UV chromatographic profile segments of 5–8 min, 13–21 min, and 23–26 min simultaneously (registered at 280 nm); and (**b**) a combination of the HPLC-UV chromatographic profile segments from 13 to 32 min obtained at 257, 280, and 316 nm, simultaneously.

Then, with the aim of improving the classification of EVOOs, several PCA models were evaluated by considering several time-window segments as above. The best results were obtained when HPLC-UV chromatographic profile segments of 5–8 min, 13–21 min, and 23–26 min (registered at 280 nm) were combined. The score plot (PC1 vs. PC2) obtained under these conditions is shown in Figure 3a. As can be seen, by employing only some specific HPLC-UV chromatographic segments, acceptable discrimination and classification among the analyzed EVOOs regarding the olive cultivar of origin was achieved. Quality control samples were clustered together and located at the middle of the model (close to the center area) showing again the good repeatability and robustness of the chromatographic and chemometric results. Discrimination along both PC axes was observed. Most of the Arbequina EVOO samples are located at the bottom-left of the plot, being clearly differentiated through the PC1 and PC2 axes against the other cultivars. Most of the Picual EVOO samples appear grouped at the top of the plot. Hojiblanca and Cornicabra EVOOs are located between the other two olive cultivars, but are still well grouped and differentiated. Obviously, complete discrimination among the analyzed samples is not achieved, as expected. For example, two Picual EVVO samples are clearly located close to where Arbequina EVOOs are clustered, while three Arbequina EVOOs are separated from their group and distributed along the areas assigned to the other three cultivars (see Figure 3a). These samples cannot be considered outliers because this behavior can be expected in this kind of study. It should be taken into account that the total amounts of extracted compounds (mainly polyphenols and phenolic acids), and their distribution in olive oils do not only depend on the olive fruit cultivar of origin but also on many other parameters such as growing area, cultivation techniques, water resources, soil management, degree of olive maturation, climate, and even the oil production method [12,13,14,15]. As a result, EVOO samples cultivated or produced under similar conditions might display some similar features and, thus, may be located in close areas. Finally, a last unsupervised PCA was performed by simultaneously combining the HPLC-UV chromatographic fingerprint profiles from 13 to 21 min obtained at the three studied wavelengths (257, 280, and 316 nm). With this model, a data matrix with a dimension of 66 samples × 4053 signals was built and subjected to PCA, and the obtained scatter plot of scores (PC1 vs. PC2) is shown in Figure 3b. The combination of these richer time-window segments obtained simultaneously at different acquisition wavelengths also allowed us to achieve an acceptable classification of the analyzed EVOO with respect to the olive cultivar of origin thanks to the discrimination among both the PC1 and PC2 axes. In this case, quality controls appeared well clustered and close to the center of the plot. Arbequina EVOOs are clustered at the left area of the plot, Picual EVOOs at the top-right section, and Hojiblanca and Cornicabra EVOOs are in the bottom area of the plot and are differentiated through the PC1 axis.

The acceptable results obtained from the exploratory PCA of the analyzed olive oils as compared to other fruit seed oils, and the analysis of EVOOs with respect to the olive cultivar of origin, suggested that the proposed HPLC-UV chromatographic fingerprints can be used as potential descriptors to achieve sample authentication and to prevent adulteration fraud in the commercialization of olive oils.

### 3.2. Authentication and Adulteration Studies by Partial Least Square Regression

The main objective of the present work is to develop a simple and reliable method to confirm the authenticity of EVOOs and to identify and quantify fraud. In the production of EVOOs, product quality is one of the main parameters guaranteeing the olive cultivar of origin, especially when product designations of origin (PDOs) are involved. However, in order to reduce production costs, EVOOs can be adulterated with cheaper fruit seed oils (sunflower, corn, etc.) or even with lower quality olive oils such as refined ones. To study this issue, a specific commercially available Arbequina monovarietal EVOO was adulterated with: (1) a commercially available Picual monovarietal EVOO, in order to evaluate whether the proposed method can guarantee PDOs of monovarietal EVOOs; (2) a commercially available ROO; and (3) a commercially available sunflower oil. In the latter two cases, the focus was on demonstrating the capacity of the proposed method to identify adulteration fraud of EVOOs with cheaper and lower quality oils. Because the difficulty of identifying and quantifying frauds increases when the adulterant is present at low concentrations, adulteration percentages from 2.5 to 85% were studied. The number of samples employed for both calibration and validation sets in the PLS studies is indicated in the experimental section. Thus, adulteration mixtures were prepared and were then analyzed with the proposed HPLC-UV method. The chromatographic fingerprints obtained at the three evaluated wavelengths were subjected to PLS. In all cases, the number of latent variables (LVs) to be used for the assessment of the models was estimated by cross validation based on both Venetian blinds with 5 splits and random blocks (4 blocks and 3 repeats). Results using the two approaches were similar.

For each one of the three adulteration cases studied, the number of LVs was estimated by depicting the latent variable number versus root-mean-square errors in cross validation (RMSECV). Then, the PLS model was built and its performance was evaluated from the scatter plot of actual versus predicted adulteration percentages. Finally, the obtained errors for both calibration and prediction steps were calculated in order to assess the overall quality of the PLS model.

**Figure 4:**
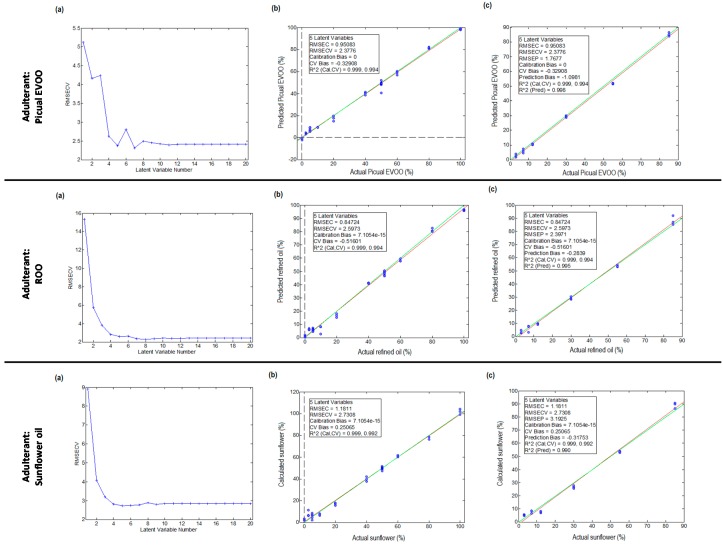
Partial least square (PLS) regression results for the adulteration of an Arbequina monovarietal EVOO using a Picual monovarietal EVOO, a ROO, and a sunflower oil as adulterants. Data set: raw HPLC-UV chromatographic fingerprints registered at 257 nm. (**a**) Estimation of the optimum number of latent variables; (**b**) validation results in the calibration step; and (**c**) validation results in the prediction step. RMSECV: root-mean-square errors in cross validation.

The first PLS model was built from raw HPLC-UV chromatographic fingerprint data without removing any signal at the three UV acquisition wavelengths. Figure 4 shows (a) the estimation of LVs; (b) the validation results in the calibration step; and (c) the validation results in the prediction step obtained in the three adulteration cases studied for HPLC-UV data registered at 257 nm.

As can be seen for the first adulteration study (with a Picual EVOO as adulterant), the lowest prediction error was attained with five LVs, so this number was chosen to quantify the Picual monovarietal percentage with respect to the adulteration of the Arbequina monovariatel EVOO sample. The agreement between the actual adulteration percentages and the predicted ones was highly satisfactory (Figure 4), with calibration errors and prediction errors (summarized in Table 1) of 0.27% and 0.25%, respectively. Similar results were achieved at the other two acquisition wavelengths (Table 1), with overall calibration and prediction errors below 0.67% and 0.41%, respectively. In the second adulteration evaluated (with ROO as the adulterant), the lowest prediction error was also attained with five LVs. Similar to the first case, the agreement between the actual ROO adulteration percentages and the predicted ones was very satisfactory (Figure 4), with calibration and prediction errors (Table 1) of 0.28% and 0.20%, respectively. These errors increased in the less favorable situation only up to 1.54% and 1.42% for the calibration and the prediction models, respectively, when the other two acquisition wavelengths were used. Finally, regarding the third adulteration study (sunflower oil as an adulterant), very similar results as compared to those previously commented were observed, with overall calibration and prediction errors below 0.77% (Table 1) for the three acquisition wavelengths.

Although the results obtained up to this point were very satisfactory, a second PLS model was built by removing from the data matrix the less discriminant HPLC chromatographic segments among the analyzed samples: the death volume segment (from 0 to 3 min) and the chromatographic cleaning and column conditioning segment (from 26 to 30 min). Thus, only the HPLC-UV chromatographic fingerprint from 3 to 26 min (including all the time-window segments discussed in the PCA section) was considered for each of the three adulterations studied. In this case, the lowest prediction error was attained with four LVs, so this number was chosen to quantify adulterant percentages. The corresponding calibration and prediction errors for all the adulteration cases studied are summarized in Table S1 (Supplementary Materials). Again, a very good agreement between the actual adulteration percentages and the predicted values was achieved, with overall errors below 2.88%.

Considering the results depicted in Table 1 and Table S1 (Supplementary Materials) we can see that, overall, data acquired at 316 nm produced higher errors (although always quite low and within the accepted parameters) in comparison to the other two acquisition wavelengths. This can be explained by the fact that data at this wavelength seems to be less informative in comparison to the other two, as was previously observed with the QCs employed in the PCA studies (see for instance Figure 1).

After the results obtained in this study, the first PLS model (employing the raw HPLC-UV chromatographic profile) with registered data either at 257 nm or at 280 nm can be proposed to carry out the identification and quantitation of fraud in EVOO samples.

## 4. Conclusions

A simple, cheap, and reliable method based on HPLC-UV has been developed to characterize and distinguish olive oils (EVOO, VOO, and ROO) from other seed-fruit oils (sunflower, corn, soy), Monovarietal EVOOs were also characterized with respect to the olive cultivar of origin, using chromatographic fingerprints and chemometrics.

Exploratory PCA showed a noticeable discrimination between olive oils and other fruit seed oils (although only slight differentiation within the latter group was achieved) when raw HPLC-UV chromatographic fingerprints (registered at any of the three acquisition wavelengths evaluated) were employed as data descriptors. In contrast, in order to achieve differentiation among monovarietal EVOOs with respect to the olive cultivar of origin (Arbequina, Picual, Hojiblanca, and Cornicabra) the selection of some discriminant time-window segments within the HPLC-UV chromatographic profile was necessary. HPLC-UV chromatographic profile segments of 5–8 min, 13–21 min, and 23–26 min (registered at 280 nm) provided acceptable discrimination among olive cultivars. Similar results were observed when simultaneously combining the HPLC-UV chromatographic fingerprint profiles from 13 to 21 min obtained at the three acquisition wavelengths.

Finally, a PLS study of the authenticity of a specific Arbequina monovarietal EVOO was performed to achieve the identification and quantitation of fraud. The sample was adulterated from 2.5 to 85% by employing three oils as adulterants: (1) Picual monovarietal EVOO; (2) a ROO; and (3) a sunflower oil. A first PLS model employing raw HPLC-UV chromatographic fingerprints without removing any signal allowed the quantification of the studied adulteration frauds with overall quantitation and prediction errors below 1.54%. A second PLS model using only HPLC chromatographic fingerprints within the time segment from 3 to 26 min provided overall quantitation and prediction errors below 2.88%. 

Certainly, some adulteration issues can be easily addressed by tracking specific markers or other families of oil components. In any case, the results obtained in this work allow us to propose HPLC-UV fingerprinting and chemometrics as a simple and efficient method for the characterization, classification, and authentication of olive oils. The method is able to guarantee olive oil monovarietal PDOs, and can also detect and quantify EVOO adulteration (minimum 2.5% adulterant) with other less expensive fruit seed oils or cheaper and lower quality olive oils such as refined ones.

## Figures and Tables

**Table 1:** Partial least square (PLS) calibration and prediction errors in the identification and quantitation of adulterants (Picual EVOO (extra-virgin olive oil), refined olive oil (ROO), and sunflower oil) in an Arbequina EVOO when using raw HPLC-UV (High performance liquid chromatography method with ultra-violet detection) chromatographic fingerprints as sample descriptors.

**Acquisition Wavelength**	**Calibration Errors (%)**		
	**Adulterant**		
	**Picual EVOO**	**ROO**	**Sunflower Oil**
257 nm	0.27	0.28	0.29
280 nm	0.19	0.33	0.37
316 nm	0.67	1.54	0.77
**Acquisition Wavelength**	**Prediction Errors (%)**		
	**Adulterant**		
	**Picual EVOO**	**ROO**	**Sunflower Oil**
257 nm	0.25	0.30	0.54
280 nm	0.41	0.33	0.48
316 nm	0.36	1.42	0.77

## Supplementary Materials

The following are available online at http://www.mdpi.com/2304-8158/7/4/44/s1. Table S1: PLS calibration and prediction errors in the identification and quantitation of adulterants (Picual EVOO, ROO, and sunflower oil) in an Arbequina EVOO when using raw HPLC-UV chromatographic fingerprints from 3 to 26 min as sample descriptors. Figure S1: HPLC-UV chromatographic fingerprints registered at 280 nm for olive oils (a,b) and sunflower oil (c). Figure S2: (a) PCA results (scores plot of PC1 vs. PC3) employing as analytical data the HPLC-UV chromatographic fingerprints obtained for time segment 14–20 min at 280 nm; (b) PCA results (score plot of PC1 vs. PC2) employing as analytical data the HPLC-UV chromatographic fingerprints obtained combining time segments of 5–8 min and 11–20 min at 280 nm. Figure S3: PCA results (score plots of PC1 vs. PC2) for the analysis of 66 EVOOs when using raw HPLC-UV chromatographic fingerprints registered at (a) 257 nm; (b) 280 nm; and (c) 316 nm as sample descriptors.
