# Supplementary material for: Authentication and Quantitation of Fraud in Extra Virgin Olive Oils Based on HPLC-UV Fingerprinting and Multivariate Calibration
Source: Foods. 2018 Mar 21;7(4):44. doi: 10.3390/foods7040044 (PMC5920409; doi:10.3390/foods7040044)
Supplement: Supplementary file 1 [file foods-07-00044-s001.pdf]

Supplementary Material:

Table S1. PLS calibration and prediction errors in the identification and quantitation of adulterants (Picual EVOO, ROO and sunflower oil) in an Arbequina EVOO when using raw HPLC-UV chromatographic fingerprints from 3 to 26 min as sample descriptors.

Acquisition wavelength	Calibration errors (%)			Prediction errors (%)		
	Adulterant			Adulterant		
	Picual EVOO	ROO	Sunflower oil	Picual EVOO	ROO	Sunflower oil
257 nm	0.12	0.47	0.23	0.10	0.26	0.55
280 nm	0.37	0.25	0.30	0.15	0.30	0.57
316 nm	2.88	1.54	0.63	0.19	1.24	0.62

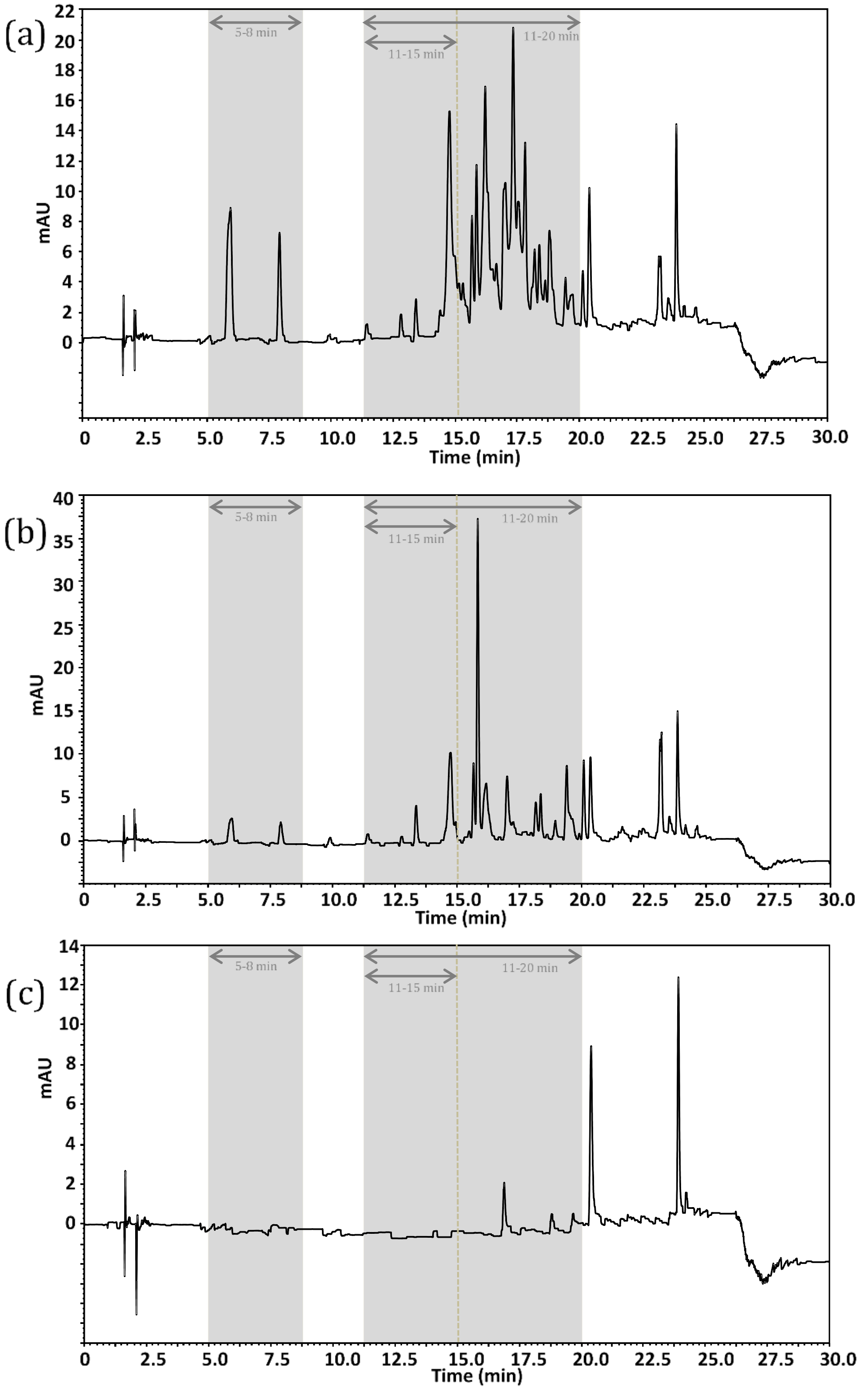

Figure S1. HPLC-UV chromatographic fingerprints registered at 280 nm of olive oils (a, b) and sunflower oil (c).

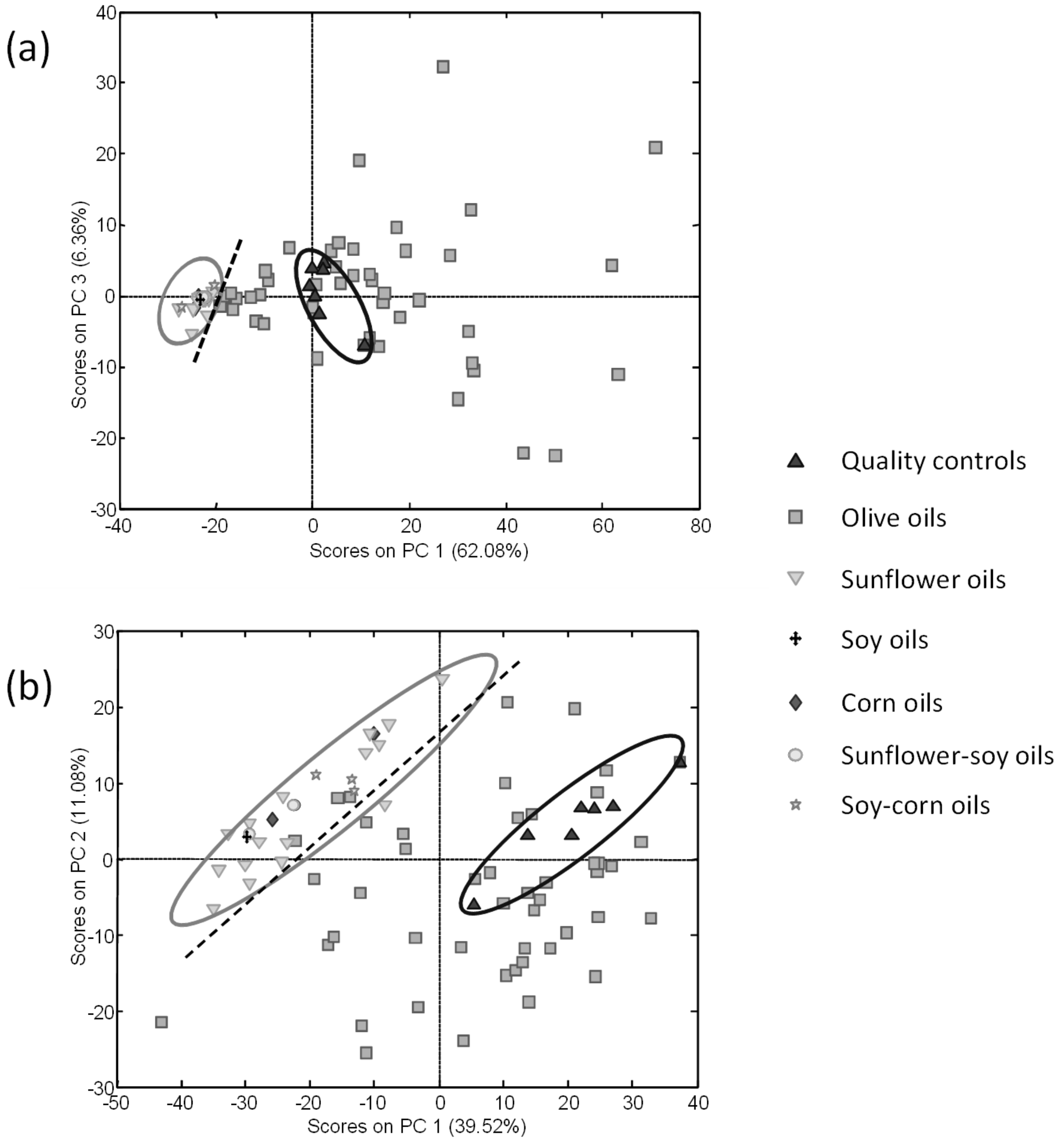

Figure S2. (a) PCA results (scores plot of PC1 *vs* PC3) employing as analytical data the HPLC-UV chromatographic fingerprints obtained for time segment 14-20 min at 280 nm. (b) PCA results (scores plot of PC1 *vs* PC2) employing as analytical data the HPLC-UV chromatographic fingerprints obtained combining time segments 5-8 min and 11-20 min at 280 nm.

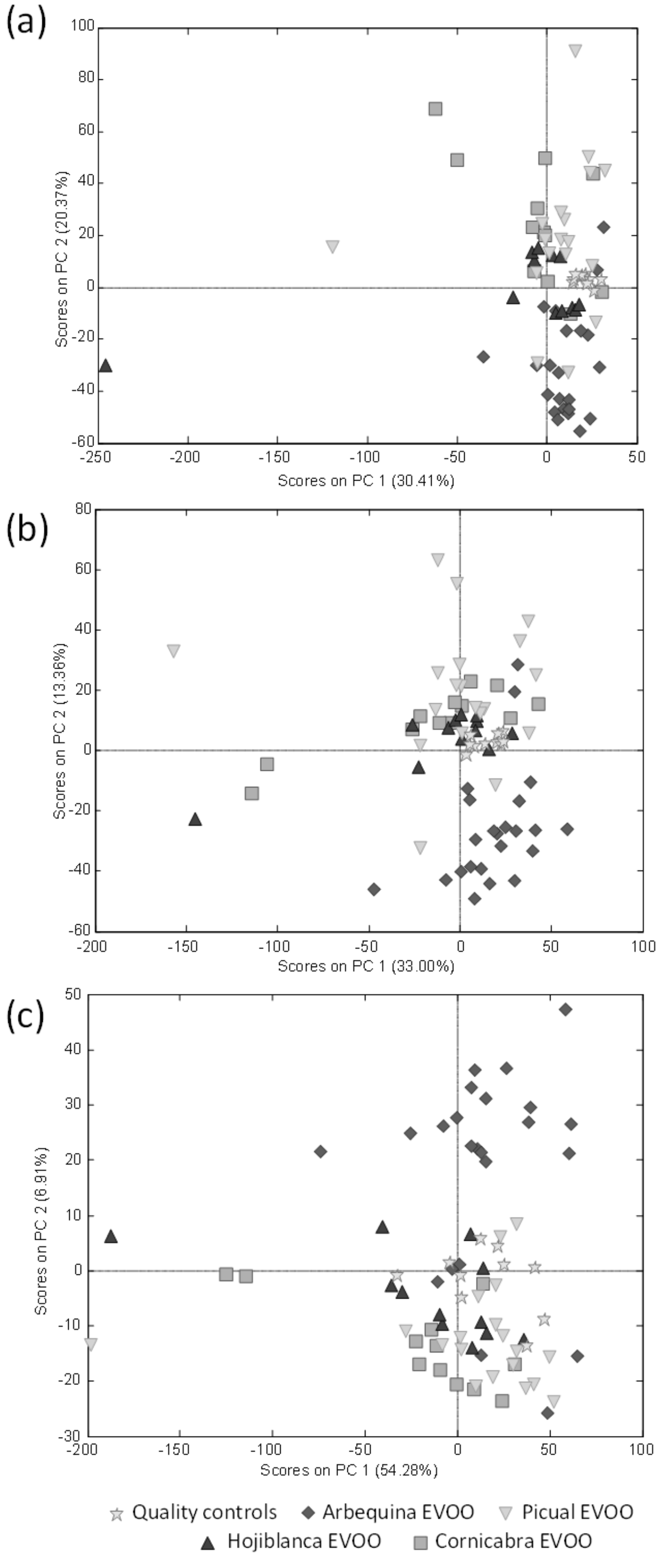

Figure S3. PCA results (score plots of PC1 *vs* PC2) for the analysis of 66 EVOOs when using raw HPLC-UV chromatographic fingerprint registered at (a) 257 nm, (b) 280 nm, and (c) 316 nm as sample descriptors.
